# Medical Cannabis Library: development of a curated database for research articles on cannabis therapeutic activity

**DOI:** 10.1186/s42238-025-00295-7

**Published:** 2025-07-07

**Authors:** Dmitry Rodin, Yael Maizels, Igor Koman

**Affiliations:** 1 Institute for Personalized and Translational Medicine, Ariel University, Ariel, Israel; 2 Institute for Personalized and Translational Medicine and Department of Molecular Biology, Faculty of Natural Sciences, Ariel University, Ariel, Israel

## Abstract

The Medical Cannabis Library (MCL) is a curated database designed to simplify the search for cannabis-related therapeutic research. Addressing the challenge of navigating vast and mixed-quality literature, the MCL consolidates over 11,000 relevant publications from Pubmed, focusing on cannabinoids as therapeutic agents. It features an advanced search interface allowing users to find information by diseases, conditions, symptoms, syndromes, and cannabinoids. The database also categorizes the relationship between cannabinoids and medical conditions as positive, negative, or neutral using natural language processing. This tool streamlines access to cannabis research, aiding professionals in understanding its therapeutic potential across various clinical indications. The MCL represents a significant advancement in organizing and accessing scientific knowledge in the field of medical cannabis.

## Introduction

Once considered a recreational drug, cannabis and its derivatives are being investigated by the medical and scientific communities as a promising treatment option for numerous conditions, including pain, multiple sclerosis, epilepsy, anxiety, depression, insomnia, nausea, seizures, and schizophrenia (Sera and Hempel-Sanderoff 2023; Blessing et al. 2015; Thiele et al. 2018; Hill et al. 2017; Crippa et al. 2018). Although there are scientific studies investigating the utility of cannabis, available data on medical marijuana is often controversial or even contradictory due to various issues, including a lack of standardization in the preparation of cannabis compounds, the wide variety of different compounds being tested, and the different effects from different cannabinoids, which sometimes oppose each other (Kansagara et al. 2017; Brown and Farquhar-Smith 2018). Despite conflicting data, the demonstrated health benefits of cannabis have accelerated its legalization. It is now decriminalized and available for medical purposes in many countries, including Canada, the US (in 38 states), and Israel (Cerdá et al. 2008; Pub. Author: EMCDDA 2017). The"new reality"that has emerged in light of marijuana legalization has generated new demands in the business, consumer, and medical communities for clear and reliable results on drug efficacy and safety. In summary, a wide body of complex research in cannabis therapeutics for a variety of indications creates a need for tools to classify and better understand this newly emerging field.

Due to increased interest in medical cannabis and its derivatives, as well as growing international legalization, there has been an explosion of scientific literature on the topic. A Pubmed search for “(cannabis OR Cannabinoid*) AND (therapy OR treatment)” returns over 25,600 search results in March 2025. This number is more than eight times that of the number in 2000 (2,866) and is expected to continue growing due to the global legalization movement and new business opportunities the cannabis field creates.

While reviewing cannabis/cannabinoids-related search queries in Pubmed we identified three main issues:

1. Many search queries on cannabinoids in Pubmed return publications that have no relation to a cannabinoid of interest. The query “Cannabigerol or CBG” in Pubmed returns 3,462 papers. However, in only 405 publications CBG refers to Cannabigerol; in other papers it is an abbreviation for other biological terms (e.g. corticosteroid-binding globulin). This issue cannot be bypassed by searching only for Cannabigerol as in this case users will lose not less than 10% of relevant publications.
2. Another issue that makes it difficult to find relevant publications is that Pubmed often returns papers where the subject of your search query is mentioned in the text body but is not a subject of the study. For example, for the same search query “Cannabigerol or CBG” 87 papers mention CBG in introduction or discussion but not in abstract, leaving us 495 publications (582 papers mentioning cannabigerol minus 87 publications where CBG presumably is not a focus of the study).
3. For most search queries, many resulting articles are reviews. However, in such an emerging field, the original research publications hold much more value. For “Cannabigerol or CBG” query at least 60 out of 582 publications are reviews.

We understand that most of these issues can be overcome by constructing correct search queries. However, this requires knowledge of how Boolean search works in Pubmed, as well as an understanding of internal Pubmed tags. In our experience, incorrect search query may result in dozens or even hundreds of unrelated publications, while missing multiple relevant publications.

Considering the overall volume of available articles in the cannabis field, the issues mentioned above significantly complicate the search process, making the analysis of resulting data a non-trivial task that would require an inordinate amount of effort and time without"external"help. New analysis tools can help scientists, professional investors, and consumers access this information faster and more effectively. Moreover, the automation of the analysis process (e.g. Natural Language Processing) may also help avoid biased searches and prevent confirmation bias, a phenomenon where a scientist focuses only on search results confirming their pre-existing hypotheses and ideas. With an increasing number of professionals and patients interested in medical cannabis, it is crucial to develop an easy-to-use tool that can sort existing data, providing structured, up-to-date, and, most importantly, unbiased information about cannabis therapeutic activity, serving as a pillar for coordinating research efforts. In this study, we present the Medical Cannabis Library (MCL), which curates knowledge from a large number of scientific papers in the field of medical cannabis and its derivatives obtained from Pubmed. The MCL enables users to search for data based on diseases, conditions, symptoms, syndromes, and cannabinoids. Consequently, users receive a list of articles relevant to a specific cannabinoid and condition. Additionally, MCL provides predictions regarding the effect of the investigated cannabinoid on the condition, allowing for further filtering. The system presented in our current study has the potential to enhance the quality of physicians'work, simplify the research process, and expedite research in the field of medical cannabis.

## Method and development

### Building comprehensive cannabinoid dictionary

First, we compiled a list of the most frequently studied cannabinoids in publications available on Pubmed. To do this, we examined hundreds of papers and online resources. We found that 74 cannabinoids had been mentioned in at least one paper on Pubmed, ranging from the well-studied cannabinoid Δ9-tetrahydrocannabinol, with thousands of publications, to dehydrocannabifuran or cannabicyclovarin, which had only been mentioned once. Pubmed does not always recognize synonyms, therefore for each cannabinoid a list of synonyms was built. For example, a search for"Δ9-THC"would return only 745 papers, while a search for “Δ9-THC OR delta9-tetrahydrocannabinol” would yield 10,055 publications. We found that Δ9-THC alone had at least 27 different name variants.

### Building initial publication dataset

In the previous step, we retrieved a list of 74 cannabinoids and their synonyms which were used to build a search query in Pubmed and create an initial dataset. Using Boolean search, we combined queries for different cannabinoids and their synonyms into a single query, and included only papers where the cannabinoid was mentioned in the Title or Abstract by utilizing the Pubmed tag [TIAB]. As we aimed to include only original studies, in the second step we filtered out all Pubmed articles with publication type"Review"or by having"Review"in the title. To simplify the text retrieval process, we used the Bio.Entrez package for Python from Biopython ver. 1.76. In total, we retrieved 25,763 publications where text abstracts were available, which were used for further analysis.

### Creating a subset of relevant publications containing diseases/syndromes/conditions and cannabinoids

As the aim of the current project was to help users find information on the therapeutic properties of various cannabinoids, we had to build a dataset of publications that studied cannabinoids in the context of one or more conditions. First, we extracted the names of cannabinoids from each paper. We utilized the Medical Subject Headings (MeSH) vocabulary, a controlled and hierarchically organized vocabulary developed by the National Library of Medicine, to construct a comprehensive list of diseases, conditions, syndromes, and symptoms. Our list comprises a total of 5,189 indications. Additionally, we included synonyms, resulting in a total of 56,997 terms. We employed this list to extract indications from the text body. For further analysis we selected publications, in which the abstracts contained at least one indication and one cannabinoid.

### Manual filtering of resulting set of publications

We manually analyzed the papers which mentioned the top-100 indications which included 90% of all publications in the dataset. From this set of 100 indications we filtered out all terms that we found too general or irrelevant (e.g. “disease”, “syndrome”, “emergencies”, etc.). We also filtered out all papers on cannabis or other drug abuse as the focus of the current project was to elucidate therapeutic properties of cannabinoids. Another step of filtration was removing all articles that do not focus their study on cannabinoids but rather study therapeutic use of cannabis as a plant or oil. The decision to exclude studies focusing on whole-plant cannabis or cannabis oil was made to ensure a precise analysis of individual cannabinoid effects. Unlike whole-plant studies, which involve complex interactions between multiple compounds, our focus was on isolating specific cannabinoid therapeutic relationships.

We discovered that the resulting subset contained 1204 unique indications among which pain, cancer, anxiety, seizures, and inflammation were the most common (Table 1).

**Table 1:** Top-10 indications in MCL

Name of condition	Number of publications
Pain	3701
Neoplasms	2244
Anxiety	1719
Seizures	1484
Epilepsy	1391
Inflammation	1371
Depression	1246
Multiple sclerosis	622
Schizophrenia	517
Catalepsy	511

The filtered dataset contained 11,441 publications. If in the same publication a cannabis compound was investigated as treatment for two different indications (e.g. depression and anxiety) the results would be split into two different studies, one with the indication “anxiety” and one with the indication “depression”. This subdivision of articles into more than one study occurred not only with indication, but also with cannabinoids, meaning if one series of experiments studied THC and other series studied CBD each series in the same article would be listed as a separate study. It is important as there were a number of publications where one cannabinoid successfully treated the indication while the other did not. In this way, we were able to present the information related to the specific cannabinoid and provide a clear picture of its effect for the specific indication. Altogether, this final result of 11,441 publications contained 48,461 studies (cannabinoid-disease pairs).

### Relation extraction between cannabinoid and indication

To accurately extract relationships between cannabinoids and indications from research publications, we employed Natural Language Processing (NLP) techniques. NLP is efficient in cannabis research because the large volume of studies and not standardized terminology make traditional search methods less effective. Unlike simple keyword searches, NLP models like BioBERT identify links between cannabinoids and medical conditions by understanding context and reducing bias. This automation improves the accuracy and efficiency of literature reviews.

To improve the applicability of our system, we incorporated a relation extraction (RE) feature to identify and classify relationships between cannabinoids and indications within each research paper. RE is a classic task of NLP, and for this purpose, we utilized BioBERT (https://github.com/dmis-lab/biobert), – a domain-specific adaptation of the BERT (Bidirectional encoder representations from transformers) model pre-trained on large-scale biomedical corpora. This model enables precise recognition of medical terms and their relationships within abstracts, allowing for the automated classification of the therapeutic effects of cannabinoids.

Our initial dataset consisted of 9,091 papers, from which we randomly selected 900 to use for model training. Among this, 110 were excluded due to insufficient abstract length, and 21 publications not in English were also filtered out leaving 769 papers for manual annotation Each paper was assigned one of the three classes: “Positive” (positive effect of a cannabinoid on indication), “Negative” (negative effect of a cannabinoid on indication), and “None” (no observed effect).

We tested various approaches for relation extraction, and we determined that the combinatorial model produced the most reliable result. This approach combined the bag-of-words features with a Convolutional Neural Network (CNN), which is a part of open-source SciSpaCy library (https://allenai.github.io/scispacy). A grid search was conducted to optimize training parameters (batch size and dropout rate). The final model was trained to classify articles into three predefined categories.

#### Training dataset preprocessing

As the first step, we replaced all detected indications and cannabinoids with words “disease” and “drug”, respectively. This masking was necessary to prevent training of the model on possible correlation between the drug/disease and the effect. Thus, we tried to force the model to “respond” only to words that describe the effect regardless of the compound or indication. Articles were then split into two sets—training set (that was used to train the model) and test set (that was used to evaluate the quality of relations extraction). The splitting was done randomly (80% to 20% for training set\ test set, respectively).

#### Quality metrics

**Fig. 1:**
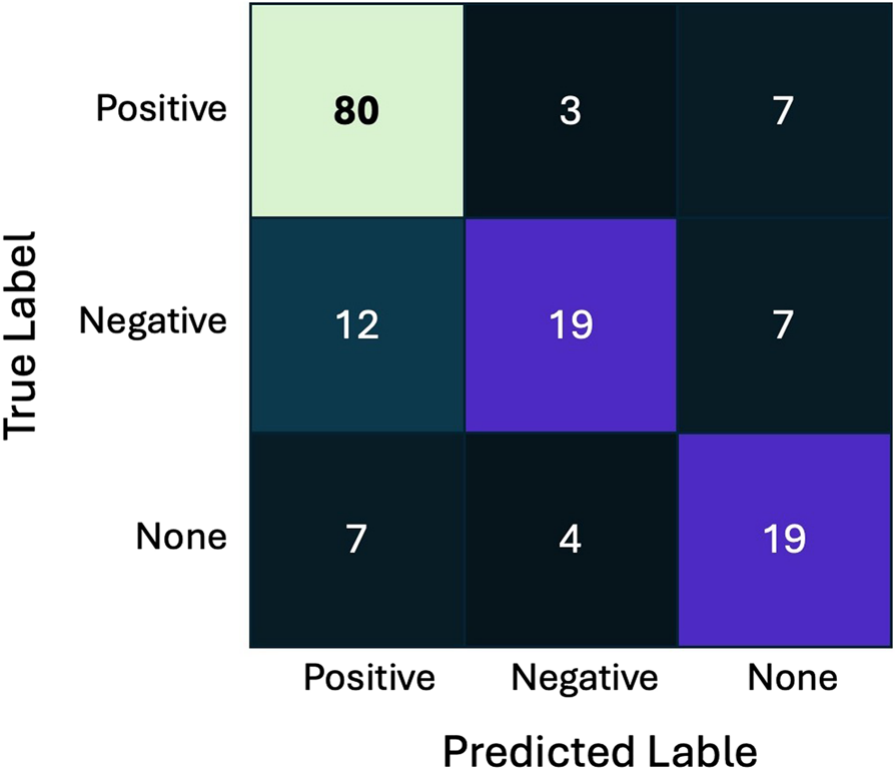
Evaluation of model quality: error matrix

To assess model quality, we used Area Under Receiver Operating Characteristic (ROC) curve (AUC). This metric is insensitive to the imbalance of classes in the sample, which allows a robust assessment of the model quality. We calculated ROC AUC between each pair of classes and averaged the resulting values. For visualization of the classification quality, we used the error matrix (Fig. 1).

The resulting accuracy of the model estimated by ROC AUC test is 87.45%.

Our model succeeded in extracting positive and neutral relations but had a higher error rate for negative relations. Since the next phase of this project involves manual validation of the extracted results, further improvements were considered unnecessary at this stage but will be implemented in the next version of the model.

Classification example:

- example = 'Our results suggest the drug could not reduce masticatory muscle disease through activating peripheral CB1 receptors.'
- Positive = 0.3245519995689392
- Negative = 0.2914993166923523
- None = 0.41434338688850403

### Building database from extracted data and creating front-end

Based on the result of data extraction using our model, we build a database summarizing all extracted data including article details (PMID, title, authors, journal and date of publication).

Technical details: an import tool was built with Laravel and MySQL to get all crucial information for sorting studies, then filters were added in PHP to return information relevant to user searches. The import tool checks incoming articles for disease synonyms so that relevant articles will be returned together by identifying in MySQL which diseases are synonyms. When a user searches for a disease, the tool will return all articles relevant to that disease as well as its synonyms. A column was also built to store the confidence level of each study vis a vis the effects of that cannabis type on each disease. We also added a filter that checks for positive or negative findings. A downloading CSV function was added to summarize and export search results using Eloquent searches and PHP's native downloading functionality. Quick links were built for terms most relevant to cannabis searches.

### Manual validation and database update

Data extracted from analyzed articles that was used to build a database is currently in process of manual validation. Despite good value in the ROC AUC test, we want to be sure that users will get as relevant results as it is currently possible. Articles that have been manually validated have confidence level 100%.

The MCL database is updated monthly through automated Pubmed searches, integrating newly published articles that meet the inclusion criteria. The system uses predefined search queries to retrieve relevant publications, which are then processed through our NLP pipeline. In addition to automated updates, a manual verification process is applied to a subset of newly added records.

## Results and summary

In our project, we implemented a number of approaches that allowed us to extract the names of cannabinoids, and indications from Pubmed research articles. We then created a tool to extract the relation between the cannabinoid and the indication. In our test set, our model has an accuracy of 87% for effect class extraction. The model was applied to classify 11,441 pubmed abstracts mentioning cannabinoids in the context of disease treatment giving us 48,461 studies (cannabinoid-disease pairs). The studies were classified into 26,450 positive case where cannabis compounds have a positive effect, 19,217 cases with negative effect, and 2794 cases where there is no effect.

As a final step, we built a user-friendly web-interface to present extracted data, results can be filtered by cannabis compound, research model and effect. Results of search queries can be analyzed online or downloaded in CSV format.

**Fig. 2:**
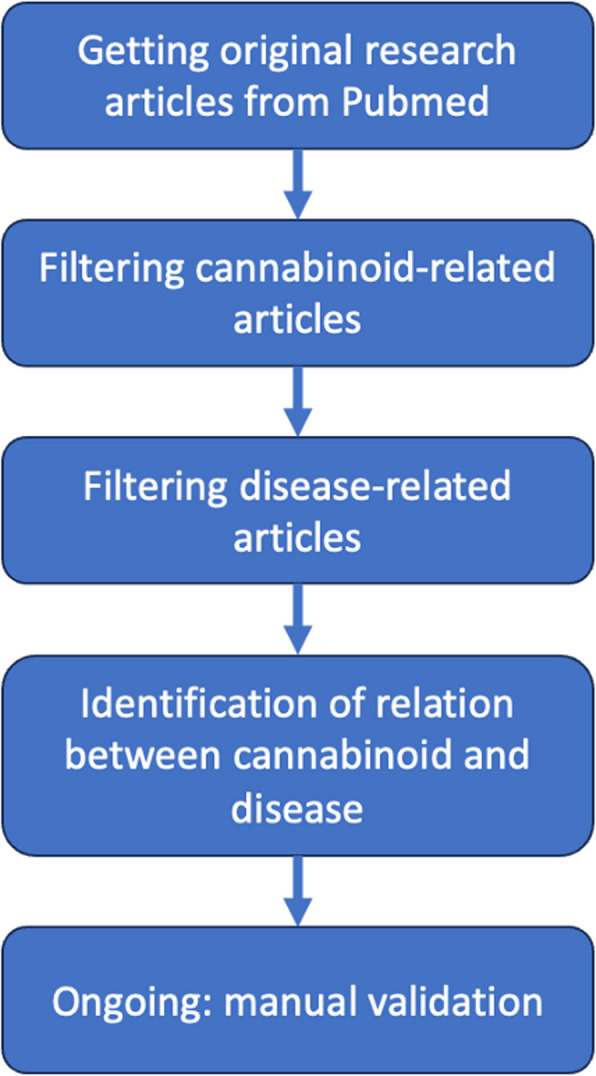
Schematic representation of the database development

Schematically, our approach for data extraction is presented in Fig. 2.

The Medical Cannabis Library is available as a service at *mcl.translation-med.com* by request.

## Discussion

MCL helps researchers access studies on the therapeutic effects of cannabinoids specifically. Unlike existing resources that focus on publication trends (Mano-Sousa et al. 2024), systematic review quality (Jugl et al. 2021), or traditional cannabis uses (Balant et al. 2021), MCL uses natural language processing (NLP) to extract and classify cannabinoid-condition relationships. This allows systematic identification of evidence on cannabinoid efficacy across medical conditions.

Bibliometric analyses show research trends but do not assess therapeutic relevance. Systematic review assessments highlight study quality but do not provide a structured, updated dataset on cannabinoid effects. Ethnobotanical databases focus on historical and cultural aspects rather than clinical applications. MCL fills these gaps by applying computational methods to organize and classify data on therapeutical applications of cannabinoids.

Unlike general AI search engines, such as Perplexity (https://www.perplexity.ai) and other LLM-based tools, MCL is designed specifically for cannabis research. While general AI tools rely on broad web and literature searches, MCL processes cannabinoid-related biomedical literature using structured NLP. It integrates BioBERT for precise entity recognition and SciSpaCy for cannabinoid-indication relation extraction.

## Limitations and future directions

Despite the advantages of NLP and ML models in processing biomedical literature, certain limitations must be acknowledged. One major limitation is the potential for data biases due to the inconsistencies in terminology across publications. For example, some studies use specific cannabinoid names, while others use broad classifications such as “phytocannabinoids.”

Another limitation is the ongoing validation process. Although our classification model achieves an accuracy of 87%, misclassifications may still occur. To address this, we have implemented manual validation for a subset of extracted relationships. So far, we have validated around 20% of the result and the process is ongoing. Future work will focus on refining search algorithms and improving automated filtering using validated relation extraction results to further enhance accuracy.

The current system primarily relies on abstracts for relation extraction. While a single abstract may not include every detail, multiple publications often examine the same topic. This increases the likelihood that missing information can be found in other abstracts. By incorporating findings from multiple abstract, the system improves the reliability and completeness of cannabinoid-indication relationship extraction without requiring full-text analysis.

We also plan to create a dedicated section for studies specifically on cannabis as a plant, extracts, or oil, to make the database more comprehensive and valuable. Additionally, we plan to integrate systematic user feedback to improve the usability and functionality of the MCL database and interface.

## Conclusion

The MCL contains a collection of articles on cannabinoid use for disease treatment. With a user-friendly web interface, it allows anyone interested in the field to search by indication and cannabinoid and gives an overall picture of all the relevant research in an unbiased, sortable, and accessible manner. This resource allows users to look at a collection of filtered articles on a topic rather than the impossible task of looking through hundreds or even thousands of articles simplifying the search of relevant scientific knowledge in the field of cannabis research. As the effects of cannabinoids are being explored in a wide variety of clinical indications this tool allows for efficient searching of the literature to get a comprehensive look at the evidence of its effect on human pathology.

The Medical Cannabis Library is currently accessible at mcl.translation-med.com – access credentials are available upon request without restrictions.

## Data Availability

All collected data is available by request at *mcl.translation-med.com.*
